# Diverse Data Sets Can Yield Reliable Information through Mechanistic Modeling: Salicylic Acid Clearance

**DOI:** 10.9734/BJPR/2015/19156

**Published:** 2015

**Authors:** G. M. Raymond, J. B. Bassingthwaighte

**Affiliations:** 1 Department of Bioengineering, University of Washington, Seattle, USA

**Keywords:** Briggs-Haldane, clearance, enzyme, half-life, JSim, Michaelis-Menten, model, reproducible, salicylic acid, salicylurate, CoA, aspirin, multilevel systems, confidence ranges

## Abstract

This is a practical example of a powerful research strategy: putting together data from studies covering a diversity of conditions can yield a scientifically sound grasp of the phenomenon when the individual observations failed to provide definitive understanding. The rationale is that defining a realistic, quantitative, explanatory hypothesis for the whole set of studies, brings about a “consilience” of the often competing hypotheses considered for individual data sets. An internally consistent conjecture linking multiple data sets simultaneously provides stronger evidence on the characteristics of a system than does analysis of individual data sets limited to narrow ranges of conditions. Our example examines three very different data sets on the clearance of salicylic acid from humans: a high concentration set from aspirin overdoses; a set with medium concentrations from a research study on the influences of the route of administration and of sex on the clearance kinetics, and a set on low dose aspirin for cardiovascular health. Three models were tested: (1) a first order reaction, (2) a Michaelis-Menten (M-M) approach, and (3) an enzyme kinetic model with forward and backward reactions. The reaction rates found from model 1 were distinctly different for the three data sets, having no commonality. The M-M model 2 fitted each of the three data sets but gave a reliable estimates of the Michaelis constant only for the medium level data (K_m_ = 24±5.4 mg/L); analyzing the three data sets together with model 2 gave K_m_ = 18±2.6 mg/L. (Estimating parameters using larger numbers of data points in an optimization increases the degrees of freedom, constraining the range of the estimates). Using the enzyme kinetic model (3) increased the number of free parameters but nevertheless improved the goodness of fit to the combined data sets, giving tighter constraints, and a lower estimated K_m_ = 14.6±2.9 mg/L, demonstrating that fitting diverse data sets with a single model improves confidence in the results. This modeling effort is also an example of reproducible science available at html://www.physiome.org/jsim/models/webmodel/NSR/SalicylicAcidClearance

## 1. INTRODUCTION

The purpose of this presentation is to demonstrate that useful information can be gleaned from the literature by putting together disparate data sets that can be integrated to yield a quantitative interpretation that could not be obtained from any of the individual data sets. We start with three clinical data sets on the clearance of aspirin from the blood. These were studies on humans, one in the range of normal therapy (10–70 mg/L, plasma levels; dose 1000 mg), one in people poisoned by overdosing (> 250 mg/L, high doses), and one on the kinetics of disappearance of the 80 mg antiplatelet dose (< 6 mg/L). The principle on which this essay is based is that we can gain mechanistic insight by analyzing the three data sets together but not from any of the individual data sets. Further, despite the fact that we use only crude, accumulated average values from different populations of patients and experimental subjects, and use no information on actual biochemical kinetics, we can present an analysis of predictive value that may be used to understand the observations and to guide therapy.

In this study, we examine data on aspirin that were gathered in three unrelated clearance studies. Thinking pharmacokinetically, one wants to interpret data in parametric terms (rate constants, enzyme affinities, membrane permeabilities, etc.) that represent the processes and provide insight into mechanism. We take advantage of the wide ranges of concentrations observed, trying to identify rate constants and affinities that should be common to the three diverse data sets.

Aspirin, acetyl salicylic acid, is rapidly hydrolyzed to acetate and salicylate. Salicylate is the effective therapeutic agent at 30 to 80 mg/L. The drug's efficacy is limited by its degradation. Mitochondrial enzymes in liver and kidney degrade salicylate to an inert product, salicylurate, that is, like salicylate itself, excreted in the urine. Binding to plasma proteins retards renal clearance of salicylate, prolonging its retention, so that most of the renal excretion is as the salicylurate. Concentrations ten times therapeutic levels are toxic, causing acidosis, and sometimes death. The LD50 (lethal dose for 50% of subjects) is about 200 mg/kg in rats and mice and probably higher in humans, i.e. of the order of more than 10 grams for a 50 kg person. Historically, fevers, pains, and inflammation were treated with preparations from willow bark from Greek times. Salicylate was explicitly identified by Stone [1]. Aspirin, acetyl-salicylate, was synthesized and marketed by Bayer in 1897, and continues to be in wide use with few problems.

## 2. OVERVIEW OF THE KINETICS

The substrate to product reaction sequence, S--> P, is:

Aspirin-->Salicylic acid -->Salicyl-CoA-->Salicylurate

The hydrolysis to salicylic acid is so fast that it can be considered instantaneous. The rate-limiting step in degradation is the conversion of salicylic acid to salicyl-CoA catalyzed by medium chain acyl-Coenzyme A synthase, and the final step is the conversion of salicyl-CoA to salicylurate, catalyzed by glycine-N-acyltransferase [2]. The conversion to salicylurate is a key step toward renal clearance, accounting for 50% [3,4] to 85% [5] of ingested salicylate. Only about 10% is urinary salicylate [6]. Presumably renal clearance by passive filtration across the glomerular membrane is zero for salicylate bound to plasma albumin, and may be slower for the ionized fraction of salicylate because of the charge. In any case the clearance basically follows the conversion to salicylurate.

We define a minimal model in which the whole body is considered as a mixing chamber into which the salicylate is absorbed, and within which an enzyme converts salicylate to salicylurate. Salicylurate is assumed to be the only product, and the only solute cleared by the kidney, ignoring the salicylate 10% mentioned just above. The converting enzymes are in mitochondria in the liver and kidney, not free in the circulation: the kinetic simplicity of the model makes sense only if the whole body mixing and the transport from blood to mitochondria are both fast compared to the conversion steps and the renal clearance.

Three models to describe and “explain” the clearance of the salicylate will be explored: (1) the first tests the concept that first order kinetics dominate the system, as if the clearance were controlled by a single exponential washout process, a totally inadequate model; (2) the second model asks if a Michaelis-Menten enzymatic reaction model can define the kinetics; (3) the third test is a more fully developed enzyme kinetic model with reversible reactions. Each model is tested against the three data sets individually and then together, asking whether or not the data from three different sets of subjects at three very different levels of dosage can be “explained” in terms of *one* structurally and parametrically self-consistent model describing the processes governing the clearance. We say “explain” in quotes since all models are incomplete and inexact.

## 3. THE DATA AND THE SOURCES

**Fig. 1:**
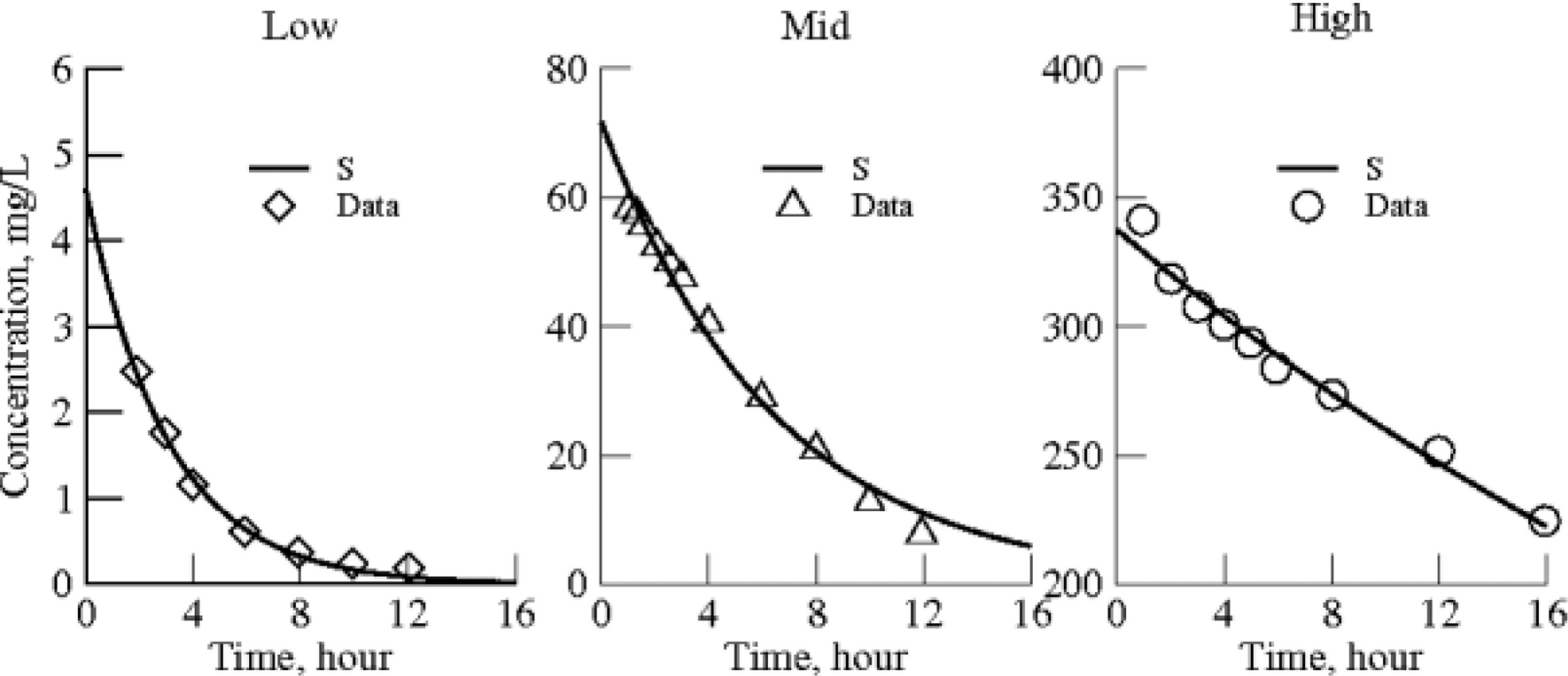
Salicylate decay curves for low, medium and high doses from three separate studies Each is a group-averaged set of data from humans; Left panel: Benedek95; middle panel: Aarons89; right panel: Prescott82. Each data set is fitted with Model 1, single exponential functions with the half-life and an initial value of concentration for each dose group as the sole parameters. Parameter values and standard deviations for the model solutions to Eq 2 are given in Table 1

The data sets are group-averaged observations on plasma salicylate concentrations as a function of time over most of a day after ingestion of aspirin. The data were published in three unrelated studies, each of which covered a different range of concentrations (Fig. 1).

The data (Fig. 1 left) for low dose aspirin ingestion [7] are the averaged plasma concentrations from ten healthy male volunteers who ingested an 81 mg tablet of aspirin. The last seven points were digitized from the authors' figure (their Fig. 1, right panel, dose period 1, open squares). “All aspects of the study were conducted in accordance with regulations of the United States Food and Drug Administration (FDA), in particular those regarding informed consent and approval by a qualified Institutional Review Board”.

The data (Fig. 1 middle) for mid dose aspirin ingestion [8] are the averaged plasma concentrations from 9 female and 9 male healthy volunteers, who ingested 1000 mg of lysine acetyl salicylate. The last eleven points were digitized from authors' Fig. 3, the solid triangles. “Each subject gave informed written consent to participate in the study and the study received the approval of an Ethics Committee”.

The data (Fig. 1, right) for the high dose aspirin ingestion [9] are the averaged plasma concentrations from 16 patients being treated for mild poisoning, salicylism, (plasma salicylate concentrations 250–400 mg/L). The last nine points were digitized from authors' figure (their Fig. 1, open circles). In these aspirin overdose cases, the authors were attempting to discern best treatment methods. The research was performed at the Regional Poisoning Treatment Centre and University Department of Therapeutics and Clinical Pharmacology, Royal Infirmary, Edinburgh EH3 9YW. “All patients were conscious and recovered uneventfully”.

## 4. METHODS OF ANALYSIS

We used a general-purpose simulation system, JSim [10], designed for data analysis, displaying results and storing them as Reproducible Exchange Packages (REP). JSim code for the three models is written in MML, Mathematical Modeling Language: the equations have the same form as one would write them on paper, with the exception that the derivative *dS/dt* is written *S:t.* Routines for solving ordinary differential equations (ODEs), partial differential equations (PDEs) and differential algebraic equations, are built into JSim and selected by the user. JSim provides 8 methods for solving ODEs, three for PDEs. Comparing different methods is a key step in code *verification*, a part of demonstrating its correctness. Eight optimizers are available for automated fitting of model solutions to data; switching from one method to another helps to determine the uniqueness of the fit. At the point of best fit both an analytical method (covariance matrix) and MonteCarlo (randomized) method are used for estimating confidence limits, and projecting uncertainty quantification. The REP provides storage of all data, figures and parameter files and retention of all the models developed for a project, in a form reproducible under Linux, MacOS X or Windows. Model code is in the APPENDICES. The models used in the analysis are installed at html://www.physiome.org and can be run over the web. They are open source and open operation, and may be downloaded from there, as can JSim itself. All data files, models, initial conditions, parameters, and resulting figures for this report are completely described in the REP file at html://www.physiome.org/jsim/models/webmodel/NSR/SalicylicAcidClearance

### 4.1 Model 1: A Descriptive First-order Washout Model

The single exponential decay or first-order process is based on the expectation that there is a single means of clearance from the body and that it is passive, whereby a constant fraction of the concentration is removed per unit time, independent of the concentration. Assuming that mixing within the body is fast compared with the rate of removal, then, using *S* for salicylate concentration mg/L, and *k* as the fraction removed per minute, the removal flux is *k* times *S*. For a given initial concentration S(t=0) = S_0_, the governing ordinary differential equation (ODE) is

(1) dS(t)/dt=−k·S(t).

The solution to this ODE is

(2) $$S(t)=S_{0}\cdot \text{exp}(-k\cdot t)$$

A semilog plot would give a straight line, a constant fraction lost per unit time. When half of the substance is gone, the solution becomes

(3) $$S_{0}/2=S_{0}\cdot \text{exp}(-k\cdot t_{\text{half}}).$$

Defining the half-life as

(4) $$t_{\text{half}}={\text{log}}_{e}(2)/k$$

allows one to rewrite Eq. 1 as

(5) $$dS(t)/dt=-({\text{log}}_{e}(2)/t_{\text{half}})\cdot S(t).$$

The descriptive parameters, k and S_0_, for the model were adjusted to fit the data by minimizing the weighted sums of square of the distances between the data points and the model solutions, the Sum of Squares of Weighted Residuals, SSWR. This can be done either manually or by automated optimization. The model code is in Appendix 1. The “import nsrunit; unit conversion on;” requests the parser (a precompiler phase) to check the equations, and any exponents or transcendental functions like sine, for unit balance [11]; the parser also inserts reconciling conversion factors, e.g. 60 sec/min, when analogous units have been used.

This is automatic, and is the first step in verifying that the model code is computing correctly, a part of JSim’s design to support the project from experiment through the steps of a VVUQ process, model development, ***V**erification* that the code computes correctly, ***V**alidation* that the model can be fitted to experimental data, and ***U**ncertainty **Q**uantification* in parameter identification, in estimation of confidence limits from the sensitivity functions or from Monte Carlo, and in making predictions.

The Model 1 parameters for initial concentrations (LS0, MS0, and HS0) and the respective rate constants, Lk, Mk, and Hk (or alternatively the half lives), may be manually adjusted to fit the data. The same process can be automated using the “Optimization” Graphical User Interface (GUI). The optimization GUI is used to automate the fitting to each data set individually to estimate the decay rate and the initial concentrations; these are reported in Table 1, along with the standard deviations estimated from the covariance matrix.

As a test to see if clearance was by a single passive first order process we optimized the model solutions to use a single decay rate to best fit the three data sets simultaneously. This idea was disproved; a single decay slope could fit only one of them reasonably well at a time. Their initial values were strikingly different, so the only situation that would give similar decay rates for the three sets of data would be if their clearances mechanisms were the same, e.g. renal clearance by glomerular filtration. This was clearly not the case, so we rejected the hypothesis of a common factor being renal passive clearance.

What we learn from this model is that decay rates are slower at high concentrations (Table 1). Raising the initial concentrations by two orders of magnitude increases the half-lives by over tenfold. The time courses of the high dose concentrations and the upper range of the mid dose concentrations are approximately linear, suggesting that a zero-order process (*S* is removed at a constant rate) might be a better model. The last points of the mid dose and all the low dose concentration-time curves are nevertheless close to an exponential decay. Putting these observations together suggests that another model, a saturable enzyme model might be better; more explicitly, a saturable enzyme model with a K_m_ somewhere between the low dose concentrations and the medium dose concentrations would make sense.

### 4.2 Model 2: The Michaelis-Menten Model for Enzymatic Reaction

The reaction sequence S--> P for salicylurate formulation is

SalicylicAcid→Salicyl−CoA→Salicylurate.

The Michaelis-Menten [12] model assumes that there is no reverse flux from salicylurate, the product *P,* back to substrate *S*. Most of the clearance of *S* follows conversion of *S* to the metabolite, P, which is cleared by the kidney.

**Fig. 2:**
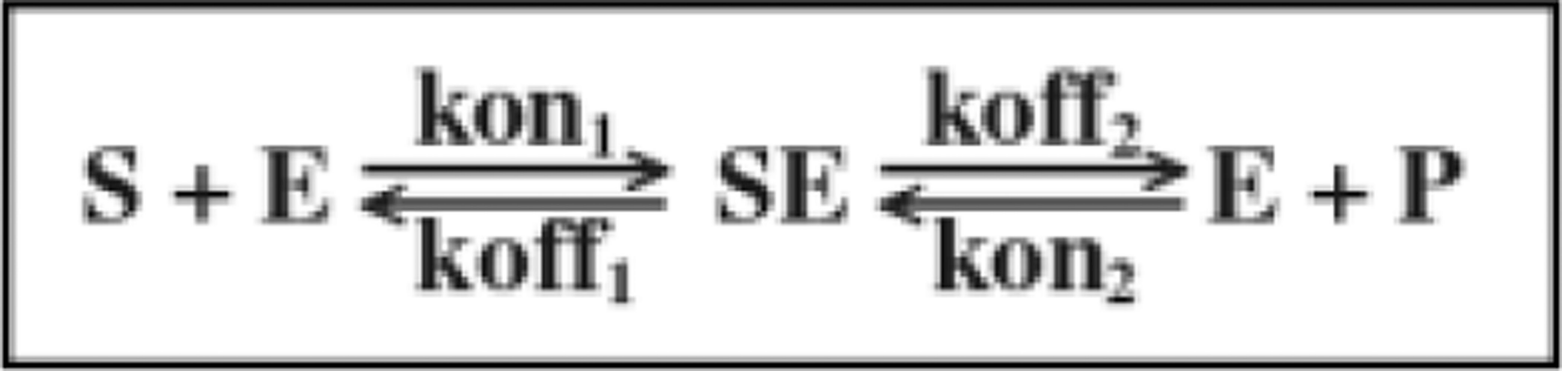
Reversible enzymatic reaction Substrate salicylate, S, combining with free enzyme, E, to form complex, SE, reacts to form product, P, and release free enzyme, E, to react with another substrate molecule

Using a single mechanistic model to fit the three data sets simultaneously should be more powerful than obtaining three independent half-life estimates, because of increasing the degrees of freedom by virtue of the constraints: more data means larger *n*, and the fewer parameters, one K_m_ and one V_max_ instead of 3 decay rates, means a tighter focus, fewer parameters per data point. Using all the data simultaneously focuses the analysis on the characteristics of an explanatory mechanism, enzymatic degradation (Fig. 2).

*S* is the substrate, salicylic acid, *E* is the enzyme, medium chain acyl-Coenzyme A synthetase, and *SE* is the enzyme-substrate complex, and *P* is still the product, salicylurate. Our assumption in this formulation is that *SE, the enzyme-substrate complex,* and *PE, the enzyme-product complex* are instantaneously interconvertible and can be regarded as the same species. The four reactions are considered to be reversible. This results in a system of four ODEs with initial conditions (Eqs. 6, 7, 8, 9, and 10) using mass balance equations

(6) $$dS/dt=-k_{\text{on}1}\cdot S\cdot E+k_{\text{off}1}\cdot SE$$

(7) $$dSE/dt=k_{\text{on}1}\cdot S\cdot E-(k_{\text{off}1}+k_{\text{off}2})\cdot SE$$

Conservation of enzyme mass allows an algebraic expression for E instead of an ODE:

(8) $$E=E_{\text{TOT}}-SE.$$

Salicylurate, P, is formed in the reaction, but is also removed by other processes, potentially by the reverse reaction with rate kon2 for backward binding to the same enzyme, or by conversion to other substances, represented by the consumption reaction at rate G:

(9) $$dP/dt=-k_{\text{on}2}\cdot P\cdot E+k_{\text{off}2}\cdot SE-G\cdot P.$$

For the most part, we will set G to zero and ignore the effect of the removal of P. The initial conditions are given as

(10) $$S(t=0)=S_{0};SE(t=0)=0;E(t=0)=E_{\text{TOT}};P(t=0)=0;$$

The Michaelis-Menten (M-M) equation is identical to the Briggs-Haldane (B-H) equation [13], but they are derived via different assumptions from Eqs 6 and 7. B-H is based on the intermediate complex, SE, being in quasi-steady state, specifically dSE/dt is small compared to the rate of change of S and P. M-M is based on the substrate, S, and the complex, SE, being in rapid equilibrium with high on- and off-rates so that the ratio E/ES is continuously defined in accord with the dissociation constant K_S_:

(11) $$K_{S}=k_{\text{off}1}/k_{\text{on}1}=E\cdot S/SE.$$

The parameter kon2 for the reversal of the product formation is assumed to be zero for the M-M model, but for the data shown is probably not true. The final equation in both cases, which we will call the Briggs-Haldane / Michaelis-Menten model (B-H/M-M), is

(12) $$dS/dt=-V_{\text{max}}\cdot S/(K_{m}+S),$$

where S is the concentration or activity of the substrate, Vmax, the maximum velocity of the reaction is given by

(13) $$V_{\text{max}}=k_{\text{off}2}\cdot E_{\text{TOT}}.$$

The substrate concentration at which the reaction velocity is half of Vmax occurs when the enzyme is half occupied, i.e. [*ES*]/ [*E_TOT_*] = 0.5, and accounts for the conversion of ES forward to P and backward to S. This defines K_m_ from Eq. 13 as

(14) $$K_{m}=(k_{\text{off}1}+k_{\text{off}2})/k_{\text{on}1}\text{ or }K_{m}=K_{S}+k_{\text{off}2}/k_{\text{on}1}$$

illustrating that when the forward, product-forming reaction is slow compare to the binding on- and off-rates that Km is only slightly greater than K_S_. When the concentration, S, is small compared to Km (as in the low dose case), the ODE for S approaches

(15) $$dS/dt\approx -(V_{\text{max}}/K_{m})\cdot S,$$

which is a first order process with the solution defining that for this situation Vmax / Km equals the k of Model 1 for the first order reaction process. When the concentration S is large compared to Km, then S / (S + Km,) approaches 1, and the ODE for S, Eq. 16, approaches

(16) $$dS/dt\approx -V_{\text{max}}.$$

This is a zero order process with solution S(t) = S_0_ − V_max_ · t. Thus the B-H/M-M model can be zero-order at high concentrations and first order at low.

The M-M code in MML is in Appendix 2. For starting values for data fitting, we estimated V_max_ using the first and last points of the high dose curve [9] to approximate Eq. 17 as

(17) $$V_{\text{max}}\approx -dS/dt\approx -\Delta SA/\Delta t\;\approx -(341mg/L-225mg/L)/(.95\text{hour}-15.94\text{hour})\approx 7.7mg/(L\cdot \text{hour}).$$

This is about 2% per hour in these aspirin-poisoned patients. From the low dose data [7] we estimate a rate constant using

(18) $$-d\text{log}(S/S_{0})/dt=V_{\text{max}}/K_{m}\approx -\partial \text{log}(S/S0)/\partial t=-\Delta \text{ log }S/\Delta t.$$

V_max_ / K_m_ = log ((2.483 mg/L)/(0.184 mg/L)) / ((12.032 hour)−1.888 hour)) = 0.257 hour^−1^, and using the estimated value for V_max_, we obtain a starting estimate for K_m_, namely, K_m_ = *V_max_*/(0.257 hour^−1^) = (7.7 mg/(L hour)/(0.257 hour^−1^) = 30.0 mg/L. This is higher than the estimate of K_m_, 16.5 mg/L, from Ho et al. [14].

**Fig. 3:**
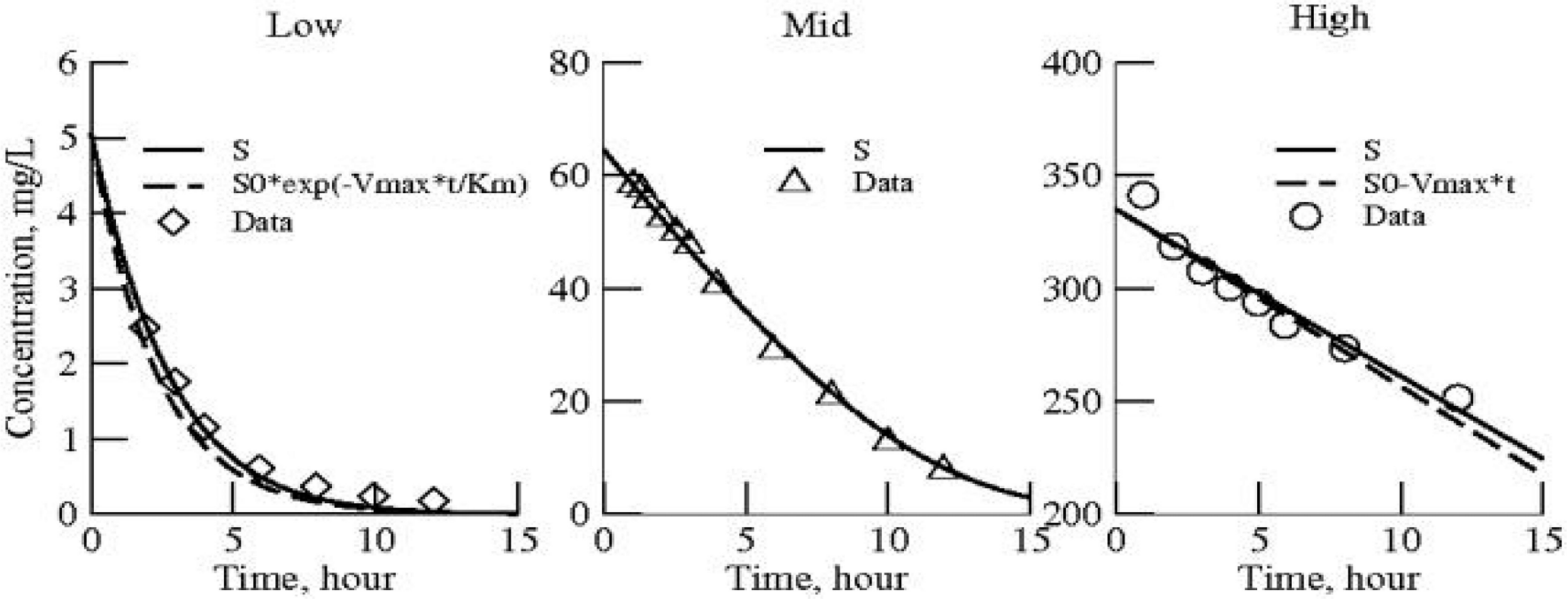
Fitting the Briggs-Haldane / Michaelis-Menten model (solid lines) to the three data sets simultaneously The five parameters are in Table 2, right column. For comparison, the dashed curve for the low dose panel represents the first order solution to the BH-MM equation, the rate constant at low concentrations being V_max_ /K_m_. The dashed line in the high dose panel is the zero order solution to the BH-MM equation, at the rate V_max_. The estimate of the K_m_, 18.2 mg/L, is more than any of the concentrations of the low dose data but less than any of those of the high dose data, meaning that its strongest influence comes from the mid dose data

For automated optimization we set the point weights to 1. For fitting the three data curves simultaneously we assigned *curve weights* that were high for the low dose data, and low for the high dose data, as stated above, so that they have similar total weight in the weighted sum of squares (Table 2, next to bottom row). The sum of squares of the differences between data and model solution for the individual data set are in the bottom row in Table 2. (A sum of the individual differences divided by the individual model point values, divided by Npoints for the individual data set, gives the fractional residual error; this is useful for comparisons amongst the individual data set fits.) The fit to the three data curves using single common values for V_max_ and K_m_ is shown in Fig. 3.

**Fig. 4:**
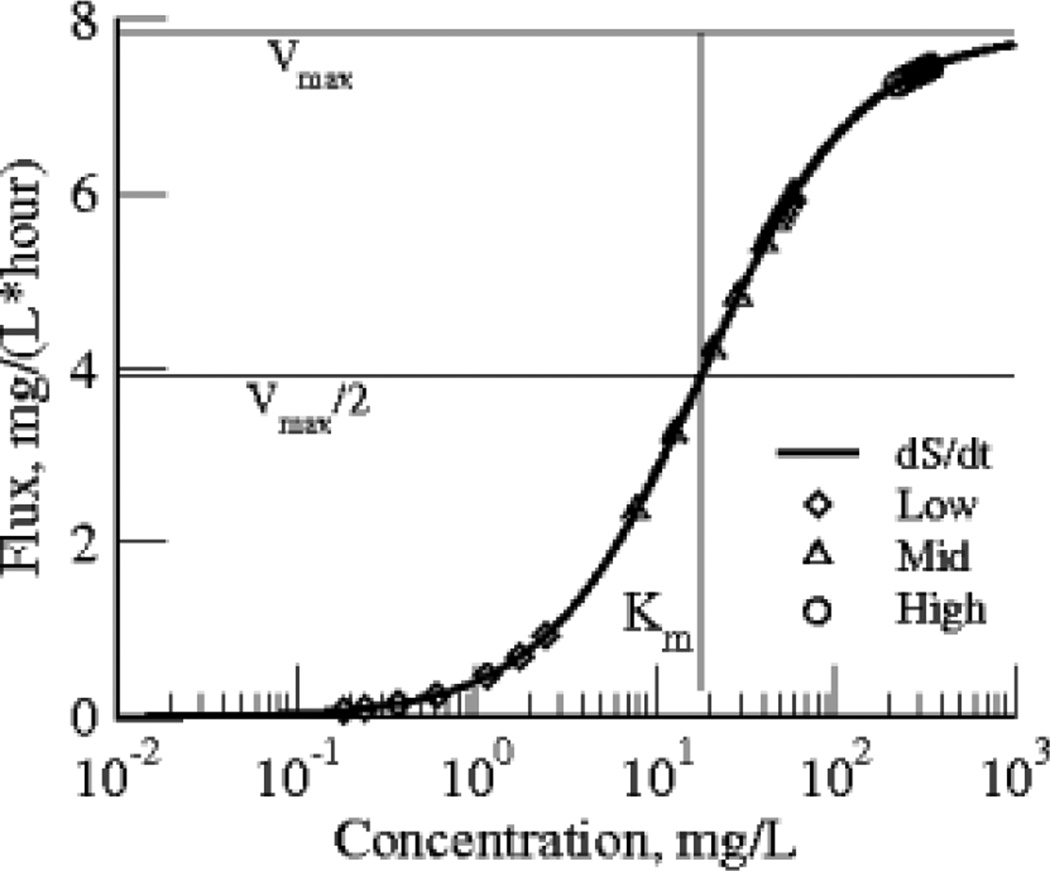
Clearance of S, dS/dt, versus S, the traditional Michaelis -Menten plot, describes the best fit of the model solution to all three data sets At V_max_ /2 the concentration S = K_m_. The intersection is at K_m_ = 18.2 mg/L, V_max_ /2 = 3.9 mg/(L*hour). The abscissa indicates the low dose data (diamonds), mid dose (triangles), and high dose (circles) mapping to the fluxes on the ordinate, just as the K_m_ maps to V_max_ /2

In Fig. 4 we plot the flux, the rate of disappearance of S (enzymatic degradation) or −dS/dt calculated from the model solutions versus the observed concentrations, the data. The intersection of this curve with V_max_ /2 gives K_m_ on the abscissa. Fluxes at the high concentrations are close to the upper limit at V_max_.

What did we learn from the irreversible M-M model? Firstly, the parameter values of this model were not well defined by fitting the individual data curves: at the low and the high concentrations the variances were worse than those for the first order washout model 1. However, when the *three* data sets were fitted with *one* set of parameters the estimates were well defined, with much smaller coefficients of variation, SD/Mean, for both the low and high concentration data. This makes for a good generality: in order to estimate K_m_ the experiment must provide data over a *wide range* and the range must encompass the K_m_. On comparing the estimates for the mid range data alone with those from the values for the three sets together, they are not statistically significantly different. The fluxes are linearly related to S/(S+K_m_); their range is greatest for the mid level data, the triangles in Fig. 4, where the ratio of flux to concentration changes steeply in the neighborhood of the K_m_. At levels below K_m_ /10 and above levels of 10 K_m_, the slopes of flux versus concentration are shallow, and therefore nearly impossible to use to estimate K_m_ accurately. Even V_max_ is poorly estimated from the high concentration data: when the enzyme is nearly saturated, with zero order kinetics, all one knows from the high dose data alone is that the concentrations are many times the K_m_.

### 4.3 Model 3: Enzyme Kinetic Model with Binding Rate Coefficients and Reversibility

The third model incorporates reactions implied in Fig. 2 and defined in Eqs 7 to 10. Since all chemical reactions are in principle reversible, the model has a reverse flux P → S, and thus allows comparing the results with those from the irreversible B-H/M-M formulation. A preliminary treatment was presented in [15]. We lack early samples that might have provided information on the rapidity of binding, so in accord with expected small solute binding to proteins we assume that k_on1_ is high, e.g. we use 3 L/(mg*sec), of the same order as fatty acids to albumin. The rate of product formation is governed by K_S_, k_off2_, and K_m_, the combination of the first two giving us K_m_ (Eq.15). The maximum forward velocity of the reaction is V_max_, which is the product E_TOT_k_off2_. From the experience with model 2 (M-M) we know that the strongest parameter estimation method is to use the three experimental data sets simultaneously, together they cover three orders of magnitude of concentrations. Not knowing the affinity K_P_ for the product, but given that reactions are reversible, the long tail of concentrations for low dose data suggest that this reaction is demonstrating its reversibility. Since P was not measured one cannot hope to obtain a unique estimate of K_P_, even though the reverse flux must occur at all three concentration levels. Analogous to K_S_ in Eq. 12, the dissociation constant K_P_ defines the equilibrium condition for *P*, *E*, and *EP* such that:

(19) $$K_{p}=k_{\text{off}2}/k_{\text{on}2}=E\cdot P/EP$$

where *EP* is considered identical to *ES* through their instantaneous interconvertibility. The model 3 code to optimize to fit the three data sets simultaneously with one set of parameters as in Appendix 3.

**Fig. 5:**
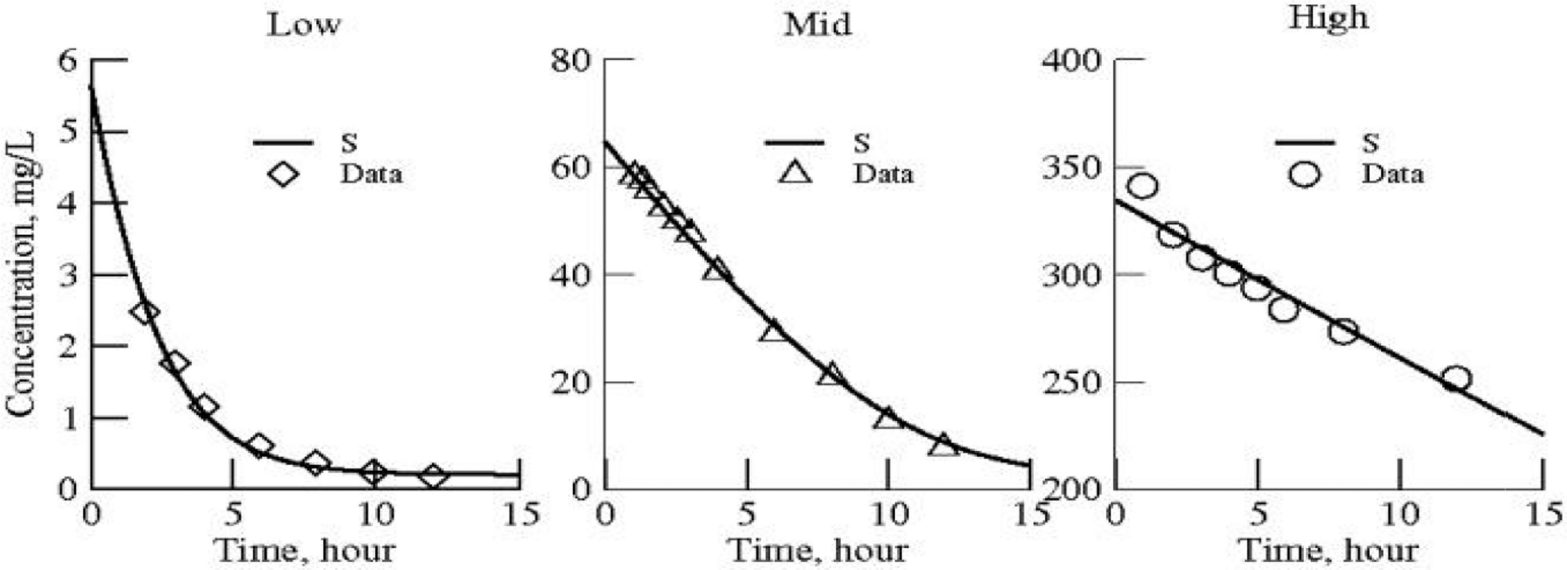
Fitting of the enzyme model solution for S(t) (heavy line in each plot) using simultaneous optimization to fit all three data sets The fitting parameters are given in Table 3, right 3 columns. The parameter values do not differ significantly between using 3 free parameters (K_s_, K_P_, and k_off2_) and using 6 free (those + the 3 initial concentrations), but the estimated confidence ranges do differ, the degrees of freedom being reduced by fixing the values of the three initial concentrations, removing them from the estimation procedure

Eqs. 7 to 10 concern the conversion of salicylic acid to salicylurate. We guesstimate *E_TOT_*, the concentration of medium chain acyl-CoA synthetase, to be 1.5e-4 mg/L, taken from the geometric mean of estimates for the human acylcoenzyme A synthetase ACSM2B, mitochondrial (ACSM2B) ELISA Kit (http://www.mybiosource.com/datasheet.php?products_id=911502). We set k_on1_ to be ~3/sec or ~10800 / hour, then optimized to estimate the remaining parameters in three different situations: (A) fitting the individual data sets, (B) fitting the three data sets simultaneously to estimate for K_S_, K_P_, and k_off2_ and the three “initial” concentrations, and (C) fixing the three initial concentrations to the values found in B and optimizing only the kinetic parameters, K_S_, K_P_, and k_off2_. From the optimized parameters we *calculated* the effective Michaelis-Menten parameters, K_m_ and V_max_, reported in Table 3 in the fourth and third rows from the bottom. The estimate of K_m_ is slightly lower than those reported by Levy [4]. The derived estimates for k_off1_, the rate of complex dissociation to produce free substrate S from the ES complex, and k_on2_, the rate of binding of product P to form EP (regarded as equilibrated with the ES form), are reported in the bottom two rows of Table 3. To obtain the estimates of the SD's for the free parameters, 1000 Monte Carlo cases were run for each combination of parameters. To do this we added 1% proportional uniform noise to each data point. Monte Carlo results were rejected when values for K_S_, K_P_, and k_off2_ were over 1000. The fits of the model solutions to the data are in Fig. 5. The low dose data are now better fitted to the tail of the curve, a result of the reverse reaction and the concentrations approaching an equilibrium level between *S* and *P*.

The decay curve for the low dose (Fig. 5) is no longer a single exponential decay, but is deviating from it by prolonging the tail: small reverse flux from *P* to *S* keeps the concentration of S above zero, so this result is substantially different from that in Fig. 3, left. The influence of the concentration of the product *P* is small because the enzyme *E* has a relatively low affinity for *P*, as indicated by the high value for K_P_ (Table 3).

With the high dose (Fig. 5, right) the decay is almost linear, showing that the enzyme is nearly saturated and that product is being formed at a rate near V_max_, a zero order process. This is much slower than the rate of decay proportional to concentration observed at low concentrations. The estimated V_max_ of 8.0 mg/(L•hr) is almost identical with the crude estimate from the slope in Eq 18.

With the middle dose the fraction of enzyme bound with S is almost constant until the concentration of *S* is less than approximately 40 mg/L. There is a gradual transition from nearly-saturated conversion initially to closer to first order clearance after 5 hours. The initial rates of removal of *S* at high and mid-level concentrations, operating close to V_max_, are described as well by the B-H/M-M model as by this model.

For these fits *E_TOT_* is not optimized since its value is more or less nominal. Its value appears in the calculations always as a part of the product, *E*_TOT_ times k_off2_. Thus *E_TOT_* and k_off2_ vary inversely with one another if both are free parameters, and in the correlation matrix would inevitably be highly correlated, in this case around *r* ~ 0.8. When covariances are high between pairs of parameters, the estimated standard deviations (SD) will be large. Without data on the concentrations of P, K_P_ is almost unconstrained. In accord with the M-M concept of rapid equilibration between E, ES and S, k_on1_ was fixed to a moderately high value of 3/sec, which then reduced the SDs of the parameter estimates to about 34% for KS and 75% for K. This illustrates that constraining the degrees of freedom narrows the parameter confidence ranges for the remaining free parameters. When only K_S_, K_P_, and k_off2_ were freely adjustable, the SD on k_off2_ shrunk to ~3%.

**Fig. 6:**
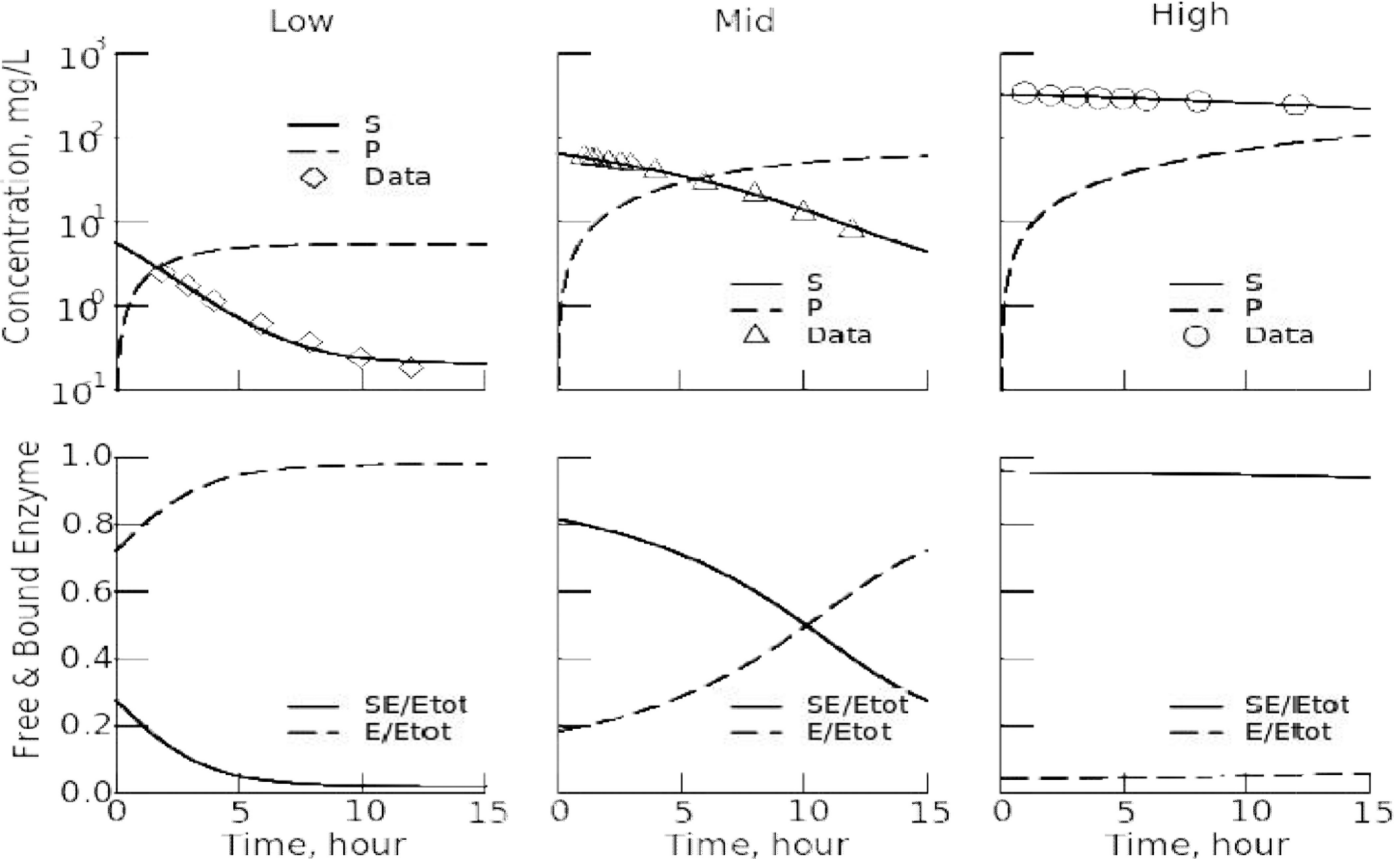
Model fitting to the three data sets simultaneously: (Parameters are in Table 3 right column) Top Panels: Data and model fits on a semilog plot, fitting S and predicting P. Lower panels: The fractional enzyme occupancy, SE/E_tot_ and free unbound enzyme E versus time, for the same three data sets as shown in the top panels. For the low dose data the enzyme is mostly free, i.e. SE/E_tot_ is low; for the high dose data, there is very little free

Fig. 6 (top panels) shows the fitting of the concentration-time curves on a semilog plot. On this scale it becomes obvious how much slower is the decay rate for the high concentrations. With the Low dose, the decay is initially almost exponential but the tail begins to level off after 8 hours: the reverse reaction, P --> S, provides an explanation for this. The middle dose data are those that give the most definitive information on K_S_: the concentrations pass from above to below K_S_, thus it makes sense that the estimates of K_S_ and k_off2_ from the middle dose data alone (column 3 of Table 3) have much narrower confidence limits than those estimated individually from the low and high doses (columns 2 and 4), and provides an estimate that is not very different from the best estimate provided by analyzing all the data simultaneously.

## 5. ENZYME SATURATION

While it is true that the fastest absolute flux, V_max_, occurs when the enzyme is completely saturated and all of the enzyme molecules are working, ES = E_tot_, *the fastest flux per unit substrate concentration* occurs when the substrate has greatest access to the enzyme, namely at low substrate concentrations, when occupancy is lowest. The idea is illustrated in Fig. 6.

With the low dose (left panel) the fraction of enzyme bound, SE/E_TOT_, was only 0.3, 30% saturated, at the beginning and fell rapidly to less than 10%. By definition, when the enzyme is unsaturated, the fraction of enzyme molecules available to bind substrate is maximal, resulting in rapid conversion of S to its metabolite, P. The fraction of free enzyme E/E_TOT_ (dotted line) rose toward close to 100% as the reaction depleted S. The mid dose panel shows that the bound fraction, the substrate-enzyme complex, SE/E_TOT_ (solid line), was initially high, over 80%, and nearly constant (quasi-steady state): the rate of change of SE/E_TOT_ was small until the concentration of S was less than approximately 40 mg/L, after which it diminished increasingly rapidly. In contrast, with the high dose (right panel) the fractional occupancy, SE/E_TOT_ is over 90 %, almost fully saturated, throughout the observation period. In this situation the fraction of the toxic substrate removed per unit time is small, about 2% per hour, and interventional therapy is desired.

## 6. DISCUSSION

The three models used to explain the disappearance of S, salicylate, from plasma, where the data came from three separate studies and spanned three orders of magnitude, were: (1) a descriptive first order decay model, chosen since it is the commonest and simplest model used in pharmacokinetic analysis, (2) a Briggs-Haldane/ Michaelis-Menten model, an approximation for an enzymatic reaction, the commonest and most frequently used model in biochemical reactions, though it is thermodynamically undefined. and (3) a thermodynamically based enzyme kinetic model with reversibility, thus fulfilling minimal thermodynamic expectations.

Model 1, first order, could only fit one data set at a time, meaning that the different data sets had unrelated clearance rates. Simultaneous optimization had no role to play since there were no parameters in common. The good individual fits with model 1 cannot be interpreted in terms of one mechanism.

The B-H / M-M formalism, model 2, fitted suitably at all doses, but only for the mid dose data did the fractional enzyme saturation, *SE/E*_tot_ shift through 50% and give a measure of the dissociation constant, K_S_. This marks a transition point between a zero order process at the earlier time to a first order process at the later time. For the low dose observations the concentrations were always less than the optimized value for K_m_, but contributed to defining it. But even though the concentrations were low, the decay did not have exactly the expected single exponential form, and instead exhibited a second slower component extending the tail of the curve, a clear deviation. The Briggs-Haldane / Michaelis-Menten model was therefore inadequate even in this region.

The “full” enzyme kinetic model, model 3, even though it does not distinguish *SE* from *PE*, and assumes that the reversibility in the pocket of the enzyme binding site occurs more or less instantaneously in either direction, properly accounts for the degree of saturation of the enzyme, so the ratio *SE/E*_tot_ is correct whether it forms from *S* or *P*. We could have used Hofmeyr's variant of the M-M model including the reversibility [16]. In a steady state they give the net forward flux, *V*_fnet_, as the difference between the unidirectional forward and the unidirectional backward fluxes:

(20) $$-dS/dt=\frac{\left(V_{f\text{max}}\cdot S/K_{s}-V_{r\text{max}}\cdot P/K_{p}\right)}{\left(1+S/K_{s}+P/K_{p}\right)}$$

where the denominator accounts for the enzyme occupancy by both *S* and *P*. The number of free parameters, four, is the same, given that we fixed k_on1_. In the high dose case particularly the denominator of Eq. 20 is large, as reflected in the slow degradation rate. This model assumes, as do the B-H/M-M models, that *E_TOT_* is small compared to *S* and *P*. In the particular cases we model, this is also true, but that restriction is not necessarily valid: in any progress curve experiment, as in the low dose case in this study, if product P were removed continuously the concentration of *S* would decrease to zero, violating the assumption that *E_TOT_* /S is small. If P is not removed, then the system would settle at equilibrium with dS/dt = 0, and the numerator of Eq 20 would go to (V_*fmax*_ · S / K_s_ = *V_rmax_* · P / K_p_), and leads to the equilibrium ratio S/P at

(21) $$\frac{S}{P}=\frac{V_{r\text{max}}}{V_{f\text{max}}}\cdot \frac{K_{s}}{K_{p}}=\frac{k_{\text{off}1}}{k_{\text{off}2}}\cdot \frac{K_{s}}{K_{p}}=\frac{k_{\text{off}1}^{2}/k_{\text{on}1}}{k_{\text{off}2}^{2}/k_{\text{on}2}}.$$

Because we have no information on concentrations of P, we have no real information on K_P_ or k_on2_, but is useful to contemplate what other circumstantial evidence might constrain the estimates. By definition, at equilibrium k_*on2*_ · K_p_ = *k_off2_*, so given the estimated values from Table 3 with k_off2_ = 14.4/sec and K_P_ = 260 mg/L, then if we think the low dose data might be reaching a constant S/P equilibrium by 15 hours one could calculate rough estimates of k_on2_ and conceivably revise the estimates of K_P_ from knowledge of the relative renal clearances of P and S.

A reason for emphasizing using the differential equations rather than the algebraic expression in Eq. 21 is that the incorporation of salicylic acid into the salicylate-CoA form is a slow reaction, violating the M-M assumption of fast binding and unbinding. Using the full equations allows this, and the present study shows that the forward reaction is partially limited by both k_on1_ and k_off2_, latter being only 5 times the former. This is important in accounting accurately for rapid changes in concentrations, obviously important in the first moments after injecting or ingesting an actively metabolized substance.

Even while arguing for the “full” model, we recognize many shortcomings our modeling, and in the data. The most obvious and worrisome is the simplification that pretends that the enzyme and the measured concentrations are in the same mixing tank, the circulating blood: the actuality is that the enzyme is really two enzymes [2] and they are not in the blood but in mitochondria of the liver and kidney. The intracellular localization means that a slow k_on1_ becomes understandable in the light of the time required for convection-permeation-diffusion processes to enter the cells and to permeate the mitochondrial membrane, and for the time to take the same route in reverse for the reaction product, salicylurate to enter the blood and to be cleared into the urine by glomerular filtration. Bloch et al. [17] also model aspirin clearance, reporting similar results with a different modeling system. To assuage our guilt feelings for not accounting for these retarding processes, we developed a crude but more general model that does that. This more complex model (SalicylateBodyMIto #377 at www.physiome.org) considers the reactants *S* and *P* and the enzyme *E* to be in the mitochondrial space, so *S* and *P* need to permeate the cell and mitochondrial membranes to exchange with a whole-body-blood-equivalent mixing chamber, in which *S* and *P* are measured, and from which both may be removed by renal clearance. Assuming fairly high permeabilities for *S* and *P*, and analyzing the data as described above, we obtained essentially similar values for K_S_, K_P_, and k_off2_ to those reported in Table 3. The conclusion is that accounting for the expected complicating and retarding influences had little effect on the identification of the key kinetics of the enzymatic process.

The assertion, “All models are wrong but some are useful,” attributed to George E. P. Box [18] is appropriate for the models and analyses presented here. There are several different paths for the metabolism of salicylates [19] with different intermediate enzymes with different V_max_'s and K_m_'s [20]. If that weren't bad enough, taking plasma concentrations from multiple subjects and averaging them seems also somewhat dubious. Although we have been successful in fitting population averages from three different studies and demonstrating that using all the data markedly improves the apparent resolution in the parameter estimates, the results remains questionable. However, the critical message from this paper is that one cannot estimate the effective K_m_ from either the low dose or the high dose data alone. One gets an approximate estimate from mid dose data. Then by using simultaneously the high mid and low dose data one gets not only confirmation of the approximation provided by the mid dose data, but increased accuracy for that estimate, presuming of course, that these different groups of humans are essentially similar. The variance of the parameter estimates are actually small compared to getting K_m_'s from general sources like KEGG, and do represent human *in vivo* conditions, so making the values particularly relevant to popular usage. Much narrower confidence ranges can be determined using isolated enzymes in test-tube experiments, e.g. as for xanthine oxidase [21] or for glycolysis [22] but the conditions are rather different from *in vivo*. There is no doubt that greater precision would be gained if we could fit detailed data from individual subjects, and account for individual characteristics such as dose per kilogram of body weight, sex of subject, age of subject, etc.; this would allow us to assess population variances meaningfully.

The analyses using B-H/M-M and the differential equations for the enzyme model both provided comparisons of the results of individual versus simultaneous analysis of the three data sets, (Tables 2 and 4) giving evidence that better estimates of parameters are obtained when the analysis is required to include all the data at once. This is virtually always the case, and is the reason for undertaking large scale, multiscale modeling of biological systems, namely to enable the systematic, simultaneous analysis of multiple data sets simultaneously on multiple components of the system. Experiments designed to provide many measures of concentrations, fluxes, conditions, temperatures, and variant perturbations of the system are important for serious modeling analyses to assess working hypotheses on a biological system.

Are the *a priori* conditions for considering the three sets of data together valid? A difficult question gives rise to an insecure answer: the groups were assayed by three different investigative teams at quite different times. But the measurement of plasma salicylate concentrations was pretty standardized by that time and reasonably accurate, well within the range of variation of the different people in each group. Probably sex differences should have been accounted for. But all were adult humans, making it reasonable to expect the variation in K_m_’s to be small compared to the huge range among species found for most enzymes in the KEGG repository. None of even the high dose group critically ill, so that their general conditions were close to normal. Our presumption that the groups were similar cannot be proven, but it would be as difficult to prove that they were not. Doubt lingers, but our judgment is that combining data sets in the analysis is more useful than not.

Extending the analysis of multiple data sets to experiments where the relationships on the data are purely statistical, and are so inaccurate that only log2-fold comparisons are near the threshold for statistical evaluation, as in mRNA array data, is *not* compatible with our approach. “The invalid assumption that correlation implies cause is probably among the two or three most serious and common errors of human reasoning.”. Stephen Jay Gould [23]. Array analyses, mRNA and protein, reveal statistical associations, not relationships, mechanisms or causation. The *a priori* condition for biophysically and biochemically-based physiological modeling analysis is that one must have firm knowledge of system connectivity and stoichiometry, which is to say cause and effect relationships amongst elements of the modeled system (the “hypothesis”). One cannot conceive, even from replicated experiments done under the same conditions providing large amounts of noisy array data, of gaining much confidence concerning the nature of associations among chemical constituents, and even less in the parameter values.

The models described here are available on the Physiome Model Repository as Model 369 at www.physiome.org. They and model 377 can be run over the web or downloaded and run on one’s own computer under the modeling system JSim. JSim can also be downloaded from the same site.

## 7. CONCLUSIONS

The modeling analysis of data, whether it is to provide descriptors or to determine mechanisms by which a system functions, gains accuracy by fitting several sets of data simultaneously with a common set of parameters. The greater the variety of good information represented by a single model the better the estimates of model parameters and the more secure the position of the model as a reasonable working hypothesis. What one seeks in biological modeling is a combination of goals: a secure description from which to make classifications; a comprehensive understanding of a self consistent system; a physicochemical system consistent with the laws of nature; an evaluation of confidence ranges for parameters and for the behavior of the system’s variables. In this study we use literature data covering a wide range of salicylate concentrations to estimate the parameters governing the clearance from the body, particularly on its enzymatic degradation. The range of the data, covering three-hundred fold in concentrations in three diverse groups, constrained the parameter estimates so that their coefficients of variation were less than 5%, a result not often achievable in clinical studies even with good experiment design, and emphasizing the power of integrating data from different sources.

## Figures and Tables

**Table 1:** Exponential decay model: Optimized parameters

Parameter value ±1 SD*	Initial concn S_0_, mg/L	Decay rate constant, fraction/hr k, hr^−1^	Half-life, min	RMS/S_0_
Low concn	4.61±0.74	0.332±0.016	126±7	0.015
Middle concn	71.8±1.75	0.156±0.008	267±14	0.026
High concn	337.5±10.3	0.026±0.002	1600±100	0.015

Optimized values for initial concentrations and rates of decay using the first order clearance model. The standard deviations of the estimates came from optimizing the group data sets individually

**Table 2:** Model 2: B-H/M-M: Parameters from optimizations Values from optimizing Briggs-Haldane/Michaelis-Menten model.

Parameter ±1 SD	Low dose individual optim	Mid dose individual optim	High dose individual optim	Simultaneous optimization
K_m_ mg/L	Fixed at 24.1	24.1±5.4	Fixed at 24.1	18.2±2.6
V_max_ mg/(L*hr)	8.4±0.49	9.2±0.89	7.7±0.56	7.85±0.38
V_max /_ K_m_ 1/hr	0.35±0.021	0.40±0.12	0.36±0.011	0.44±0.07
LS_0_ mg/L	5.07±0.23	-	-	5.07±0.48
MS_0_ mg/L	-	65.99±0.57	-	64.59±0.99
HS_0_ mg/L	-	-	333.1±1.6	335.0±2.5
Wgt in Simult Optimization	1.6435	0.0211	0.0034	Curve Wgts at left
Σ|S'_i_−S_i_|/S'_i_/N_p_	0.170	0.014	0.017	0.096

The shaded regions mark estimates of parameters with such low sensitivities that the results had no meaning until we decided to use the estimate of K_m_ from the mid dose data as fixed values of K_m_ and optimized on that basis. If the calculations for the shaded boxes were chosen in accord with the K_m_ from the simultaneous fit (right column) the Vmax would change in proportion and V_max_ / K_m_ would not change. The SD for V_max_ / K_m for_ simultaneous optimization was calculated by a Monte Carlo iterative optimization using the MML function for setting the five parameters (Vmax, K_m_, and the three S_0_'s) defined by their individual Gaussian probability density functions, doing 10^4^ optimizations. The SD for V_max_/K_m_ for the mid dose data was found by using Caladis (www.Caladis.org/compute/), also a Monte Carlo method. Error assessments are provided in the bottom row, the fractional error per point, ΣS'_i_−S_i_|/S'_i_/N_p_

**Table 3:** Enzyme kinetic model: Estimated parameters under three conditions Estimated parameter values for the enzyme model

	A. Optimizing to fit individual data sets at each dose level			B. Simultaneous optimization to fit all three dose level data using 6 free parameters			C. Simultaneous optimization to fit all three dose levels with S_0_ fixed		

Parameter	Low dose ±1 SD	Medium dose ±1 SD	High dose ±1 SD	How obtained	Estimate ±1 SD	SD/ Mean	How obtained	Estimate ±1 SD	SD/ Mean
K_S_ mg/L	6.33±2.10	8.30±2.54	181±131	Opt	11.9±0.66	±5.5%	Opt	10.44±0.18	±1.7%
K_P_ mg/L	169±67	246±130	91±68	Opt	335±30.4	±9.1%	Opt	273.5±6.5	±2.4%
k_off2_ 1/sec	7.45±1.96	14.7±0.67	55.4±30.4	Opt	15.4±0.29	±1.9%	Opt	14.82±0.13	±0.9%
LS_0_ mg/L	4.57±0.12	--	--	Opt	5.39±0.09	±1.7%	Fixed	5.67	--
MS_0_ mg/L	--	65.8±0.25	--	Opt	65.5±0.22	±0.3%	Fixed	64.9	--
HS_0_ mg/L	--	--	353±6.0	Opt	336±0.98	±0.3%	Fixed	335	--
k_on1_ L/(mg•sec)	3.0	3.0	3.0	Fixed	3.0	--	Fixed	3	--
*E*_TOT_ mg/L	1.5e-4	1.5e-4	1.5e-4	Fixed	1.5e-4	--	Fixed	1.5e-4	--
K_m_ mg/L = K_S_ + k_off2_ / k_on1_	8.82±2.75	13.2±2.76	199±131	Calc	17.1±0.75	±4.4%	Calc	15.4±0.22	±1.4%
V_max_ mg/(L•hr) = _TOT_•k_off2_	4.02±1.06	7.94±0.36	29.9±16.4	Calc	8.30±0.15	±1.9%	Calc	8.00±0.07	±0.9%
k_off1_ 1/sec = K_S_•k_on1_	19.0±6.3	24.9±7.6	542±393	Calc	35.8±1.99	±5.5%	Calc	31.3±0.55	±1.7%
k_on2_ L/(mg•sec) = k_off2_/K_P_	0.0467±0.0064	0.077±0.044	6.4±20.5	Calc	0.046±0.0042	±9.2	Calc	0.054±0.0014	±2.5%

Assuming a fixed value for kon1 = 3 L/ (mg• sec), we used three approaches: [A] (columns 2 to 4): “Unconstrained” fitting of the three data sets individually; [B] (Columns 5 to 7): Simultaneous fitting of the three data sets using K_S_, K_P_, and k_off_2, and the three initial values, S(t=0). [C] (Columns 8 to 10): Simultaneous fitting of the three data sets using only K_S_, K_P_, and k_off_2, with all other parameters fixed. The resulting fit is displayed in Fig. 5
